# Redundant Roles of Rpn10 and Rpn13 in Recognition of Ubiquitinated Proteins and Cellular Homeostasis

**DOI:** 10.1371/journal.pgen.1005401

**Published:** 2015-07-29

**Authors:** Jun Hamazaki, Shoshiro Hirayama, Shigeo Murata

**Affiliations:** Laboratory of Protein Metabolism, Graduate School of Pharmaceutical Sciences, The University of Tokyo, Bunkyo-ku, Tokyo, Japan; Harvard Medical School, United States of America

## Abstract

Intracellular proteins tagged with ubiquitin chains are targeted to the 26S proteasome for degradation. The two subunits, Rpn10 and Rpn13, function as ubiquitin receptors of the proteasome. However, differences in roles between Rpn10 and Rpn13 in mammals remains to be understood. We analyzed mice deficient for Rpn13 and Rpn10. Liver-specific deletion of either Rpn10 or Rpn13 showed only modest impairment, but simultaneous loss of both caused severe liver injury accompanied by massive accumulation of ubiquitin conjugates, which was recovered by re-expression of either Rpn10 or Rpn13. We also found that mHR23B and ubiquilin/Plic-1 and -4 failed to bind to the proteasome in the absence of both Rpn10 and Rpn13, suggesting that these two subunits are the main receptors for these UBL-UBA proteins that deliver ubiquitinated proteins to the proteasome. Our results indicate that Rpn13 mostly plays a redundant role with Rpn10 in recognition of ubiquitinated proteins and maintaining homeostasis in *Mus musculus*.

## Author Summary

At least two major ubiquitin receptor subunits that directly capture ubiquitin chains have been identified in the proteasome: Rpn10 and Rpn13. Analyses in *Saccharomyces cerevisiae* have suggested only a modest role of Rpn10 and Rpn13 in the recruitment of ubiquitinated proteins, as double deletion of Rpn10 and Rpn13 causes very mild phenotypes. Considering that ubiquitin recognition is an essential process for protein degradation by the proteasome and that failure in degradation of ubiquitinated proteins leads to human diseases such as neurodegeneration, it is important to evaluate the role of Rpn10 and Rpn13 in mammals. Liver-specific deletion of either Rpn10 or Rpn13 showed modest impairment, but simultaneous loss of both Rpn10 and Rpn13 caused severe liver injury accompanied by massive accumulation of ubiquitin conjugates and failure in recruiting mHR23B and ubiquilin/Plic-1 and -4 proteins, which deliver ubiquitinated proteins to the proteasome. Our findings indicate that the largely redundant roles of Rpn10 and Rpn13 in ubiquitin recognition and recruitment of mHR23B and ubiquilin/Plic-1 and -4 are essential for cellular homeostasis in mammals and should provide information for understanding the mechanism of ubiquitin recognition by the 26S proteasome in mammals and for development of therapeutic agents targeting protein degradation.

## Introduction

The ubiquitin-proteasome system is the main non-lysosomal proteolytic pathway through which regulatory proteins and misfolded proteins are degraded in eukaryotic cells [1,2]. Ubiquitin chains are covalently attached to target proteins through the coordinated effort of an enzymatic cascade. Ubiquitinated proteins are then recognized and degraded by the 26S proteasome in an ATP-dependent manner. The 26S proteasome is composed of one proteolytically active 20S core particle (CP) and 19S regulatory particles (RP) attached to one or both ends of the CP [3].

The RP plays an essential role in the degradation of ubiquitinated proteins by recognizing ubiquitin chains, deubiquitinating and unfolding substrate proteins, opening the gate of the CP, and translocating the substrates into the CP. The RP can be divided into two subcomplexes; the base and the lid [2]. The base contains six ATPase subunits Rpt1–Rpt6 and two large non-ATPase subunits Rpn1 (Q8VDM4) and Rpn2 (Q3TXS7), which function as scaffolds for molecules that modulate proteasome functions, such as Rpn13 (encoded by *Adrm1* [56436] in mice), Uch37 (Q9WUP7), and Usp14 (Q9JMA1) [4–8]. The lid has been shown to be essential for the degradation of ubiquitinated proteins through the function of Rpn11 (O35593), which cleaves ubiquitin (P0CG50) chains from substrates prior to degradation [9,10].

The RP has two major ubiquitin receptor subunits, Rpn10 (P38886) and Rpn13 (O13563), which directly bind to ubiquitin chains [11–13]. Rpn10 and Rpn13 can also receive ubiquitinated proteins from extraproteasomal UBL-UBA proteins, such as HR23 (P54728), ubiquilin (also called Plic) (Q8R317, Q99NB8), and Ddi1 (Q9DAF3), which have been reported to bind to either Rpn1, Rpn10, or Rpn13 via ubiquitin-like (UBL) domains and to ubiquitin chains via ubiquitin-associated (UBA) domains [14,15].

Rpn10 is composed of an N-terminal von Willebrand factor A (VWA) domain and a C-terminal ubiquitin interacting motif (UIM). While *S*. *cerevisiae* Rpn10 has a single UIM that preferentially binds to K48-linked ubiquitin chains [16], human Rpn10 has two UIMs and binds to both K48-and K63-linked ubiquitin chains with equally high affinities by using the two UIMs in a cooperative manner [17–20]. Previously, we demonstrated that mice (*Mus musculus*) lacking the UIMs of the Rpn10 (Rpn10ΔUIM) exhibited embryonic lethality, suggesting the importance of UIMs in Rpn10-mediated recognition of ubiquitinated proteins [21]. Rpn13 has an N-terminal pleckstrin-like receptor of ubiquitin (PRU) domain, which binds preferentially to the proximal ubiquitin of K48-linked diubiquitin chains [13]. Rpn10 and Rpn13 can bind simultaneously to a K48-linked diubiquitin; Rpn13 binds to the proximal ubiquitin while Rpn10 binds to the distal one [20]. However, it is unclear whether such coordination of Rpn10 and Rpn13 occurs *in vivo*.

The recognition pathways for ubiquitinated substrates appear to have diverged in different species. For example, neither Rpn10 nor Rpn13 is essential in *S*. *cerevisiae* [12,22]. However, Rpn10 is essential in mice and *Drosophila melanogaster* [21,23]. In addition, Rpn13-null mice carrying a gene trap mutation were smaller at birth and infertile due to defective gametogenesis [24]. Similarly, the UBL-UBA proteins are not essential for *S*. *cerevisiae* cell growth, while some have been shown to be essential in mouse development [12,25–27]. Although both Rpn10 and Rpn13 are considered major receptors for direct recognition of ubiquitinated substrates by the 26S proteasome, the biological significance of Rpn13 and detailed mechanisms of recognition of ubiquitinated proteins by these two receptors are still not fully understood [14,28].

In this study, to examine the recognition pathway for ubiquitinated substrates in mice, we generated Rpn13-null mice and liver-specific Rpn13-deficient mice. Rpn13-null mice died soon after birth. We also revealed that the deletion of both Rpn10-UIM and Rpn13 in the liver caused significant accumulation of ubiquitinated proteins due to impaired recognition of ubiquitinated proteins and defects in recruitment of mHR23B and ubiquilin/Plic-1 and -4 to the proteasome. Our results indicate that the largely, if not entirely, redundant roles of Rpn10 and Rpn13 in ubiquitin recognition and recruitment of mHR23B and ubiquilin/Plic-1 and -4 are essential for cellular homeostasis in mammals.

## Results

### Loss of Rpn13 causes neonatal lethality in mice

**Fig 1:**
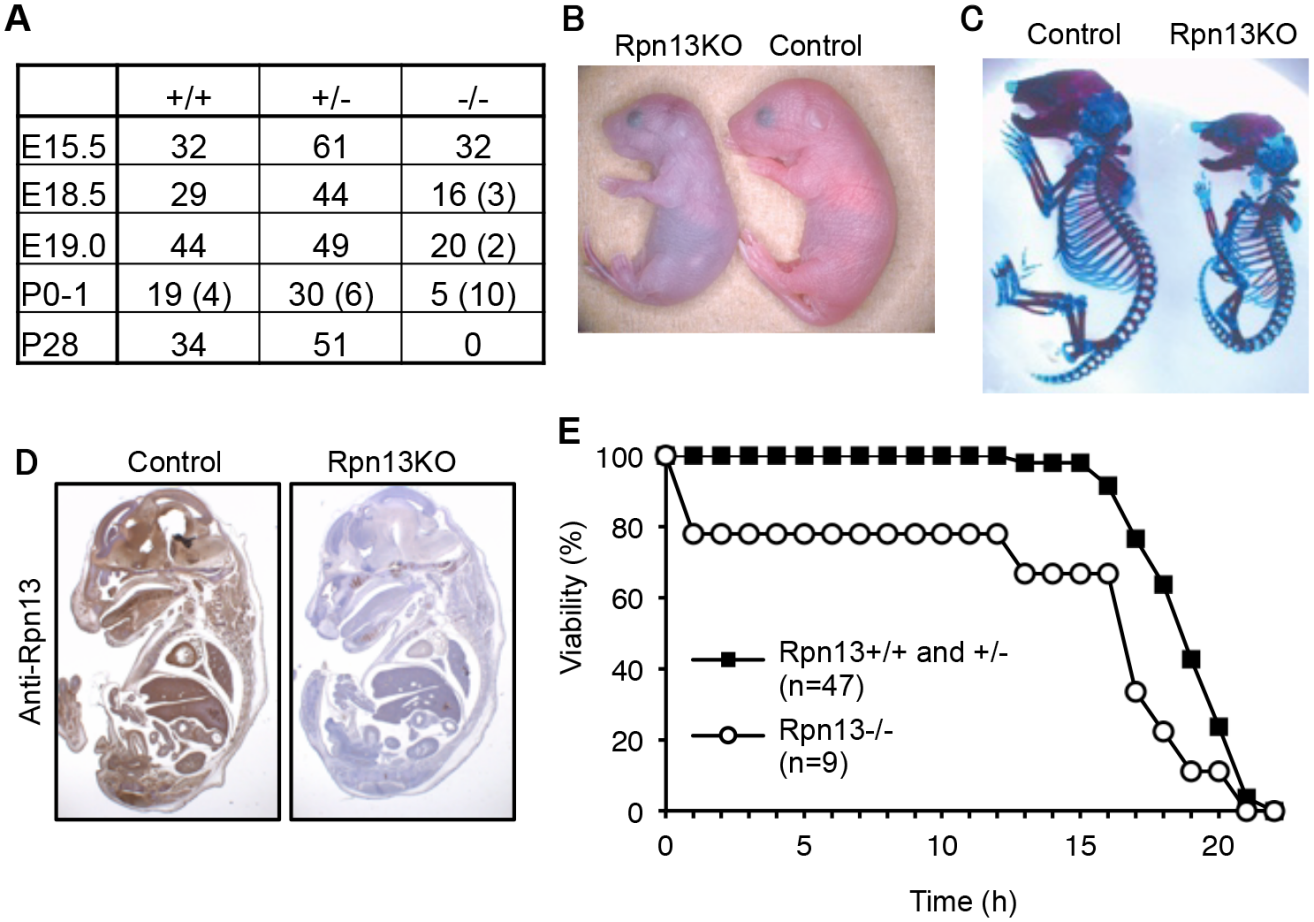
Loss of Rpn13 causes neonatal lethality in mice. (A) Genotype frequencies of embryos produced from *Adrm1* ^*+/-*^ (*Rpn13* ^*+/-*^) mouse intercrosses. Numbers in parenthesis indicate resorbed fetuses or dead newborns. E: Embryonic day, P: Postnatal day. (B) Gross appearance of control and Rpn13KO littermates shortly after birth. (C) Skeletal analysis of E18.5 littermates by Alzarin red (bone) and Alcian blue (cartilage) staining. (D) Immunohistochemical analysis of sagittally sectioned E18.5 littermates by Rpn13 antibody. (E) Survival curves of newborn mice. Control and Rpn13KO mice were delivered by cesarean section.

Rpn13 is encoded by *Adrm1* in the mouse genome. A targeting vector was designed to modify the *Adrm1* gene by homologous recombination in ES cells so that exon 3 and exon 4 of the gene were flanked by *loxP* sites (S1 Fig). The floxed allele was confirmed by Southern blot analysis (S1 Fig). Deletion of exon 3 and exon 4 of the gene by expressing DNA recombinase Cre causes a frame shift and emergence of a stop codon in exon 5. Therefore, even if splicing occurred between exon 2 and exon 5, the function of Rpn13 would be disrupted. To obtain mice lacking Rpn13 (Rpn13KO), *Adrm1* ^*F/F*^ mice were crossed with mice expressing Cre recombinase throughout the body under the control of the adenovirus-derived EIIa promoter [29]. Mice heterozygous for *Adrm1* (*Adrm1* ^*+/-*^) were healthy, fertile, and did not show any obvious phenotypes. Intercrosses of *Adrm1* ^*+/-*^ mice revealed that Rpn13KO mice were delivered at lower frequency than the expected Mendelian frequency (Fig 1A). The body weight of *Adrm1* ^*-/-*^ neonates (0.86 ± 0.13 g, n = 9) was significantly smaller than that of *Adrm1* ^*+/+*^ and *Adrm1* ^*+/-*^ neonates (1.07 ± 0.11 g, n = 47; p < 0.01), and all *Adrm1* ^*-/-*^ neonates were runted, cyanotic, did not move spontaneously, and died within 1 day after birth (Fig 1B). Eventually, no *Adrm1* ^*-/-*^ mice were obtained at postnatal day 28 (P28) (Fig 1A). These results indicate that a Rpn13 homozygous mutation causes postnatal lethality.

To understand the cause of postnatal death, we examined the skeletal structure of P0 mice (Fig 1C), whole body sections of embryonic day 18.5 (E18.5) embryos (Fig 1D), histology of the placenta of E18.5 and E15.5 embryos (S2 Fig), and the heart of P0 neonates (S2 Fig). We confirmed loss of Rpn13 protein in a whole body section (Fig 1D). However, we were not able to detect apparent morphological and histological defects in organs of Rpn13KO mice other than their small sizes. Since newborn Rpn13KO mice were cyanotic and did not show an abnormality in the heart that would cause congestion (S2 Fig), we performed histological analysis of the lung. At E18.5, there was no difference between control and Rpn13KO mice (S2 Fig). However, pulmonary alveoli of Rpn13KO newborns did not expand, while those of control littermates did (S2 Fig). This result indicates that Rpn13KO mice failed to breathe after birth. Because Rpn13KO neonates appeared to be breathing in response to mechanical skin stimulation, we tested whether cesarean delivery followed by stimulation for breathing would rescue the early death of Rpn13KO neonates. Most Rpn13KO neonates delivered by cesarean section survived more than ten hours, which is longer than the survival time of Rpn13KO neonates obtained by natural delivery, yet shorter than that of control littermates (Fig 1E). Histologically, these stimulated Rpn13KO neonates showed expanded alveoli, similar to control littermates (S2 Fig). These results suggest that one of the major causes of the early neonatal death of Rpn13KO mice is spontaneous breathing failure.

### Rpn13 deficiency in the liver impairs degradation of ubiquitinated proteins

**Fig 2:**
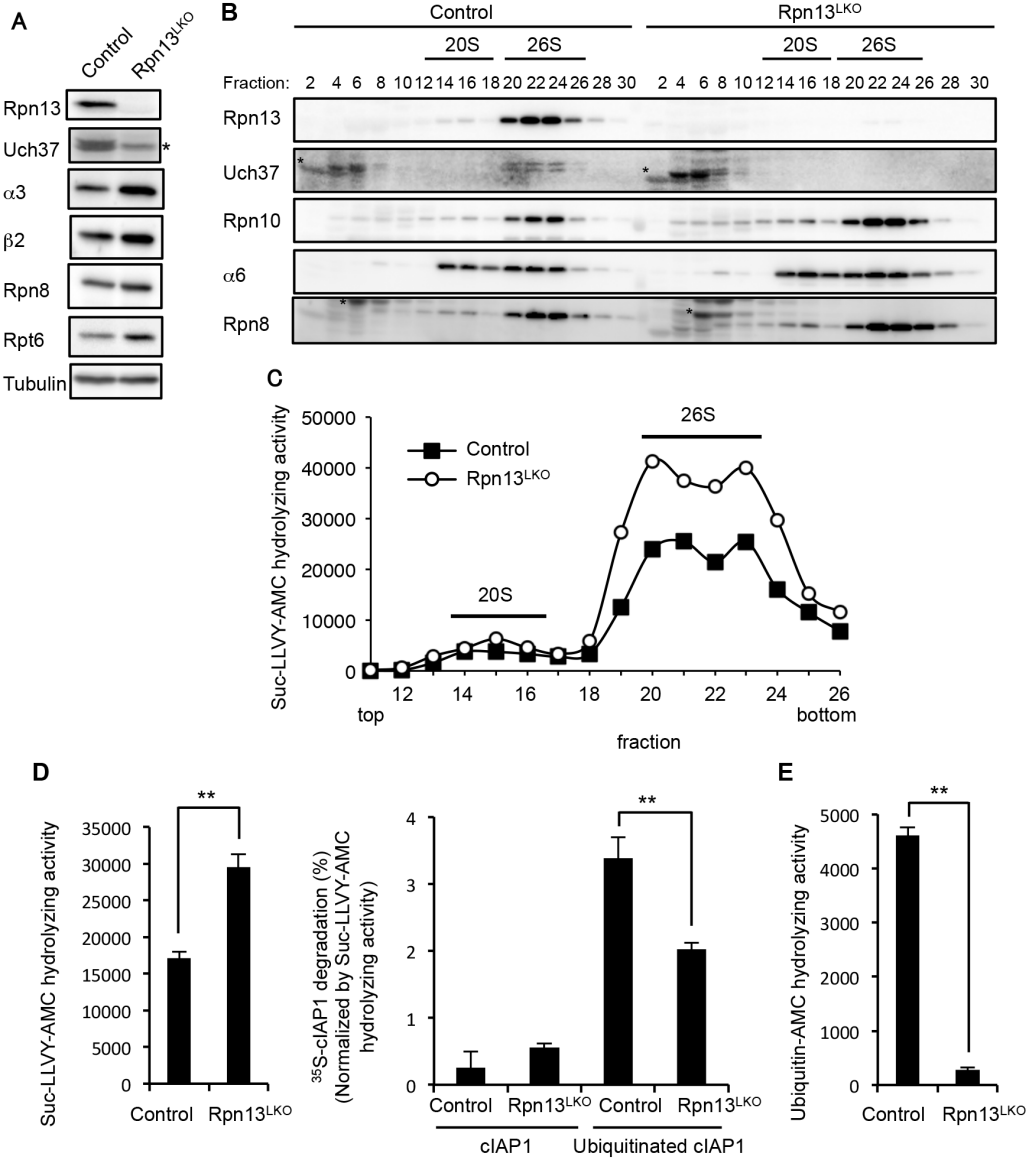
Rpn13 deficiency in the liver impairs degradation of ubiquitinated proteins. (A) Immunoblot analysis of liver lysates from 8-week-old control and Rpn13^LKO^ mice with antibodies against the indicated proteins. Asterisk indicates a nonspecific band. (B) Lysates from control and Rpn13^LKO^ livers were fractionated by glycerol gradient centrifugation (8 to 32% glycerol from fraction 1 to 30) and an equal amount of each fraction was used for immunoblot analysis using antibodies against the indicated proteins. Asterisks indicate nonspecific bands. (C) Each fraction of (B) was assayed for chymotrypsin-like activity using Suc-LLVY-AMC as a substrate. (D) The 26S proteasome fractions of (C) (fractions 20–23) were subjected to the assay of chymotrypsin-like activity (left panel), and degradation of ^35^S-labeled cIAP1 with or without ubiquitination was measured and normalized by chymotrypsin-like activity (right panel). Data are mean ±standard deviations from triplicate experiments. **p < 0.01. (E) The deubiquitinating activities of 26S proteasome fractions of (C) were measured using ubiquitin-AMC as a substrate. Data are mean ± standard deviations from triplicate experiments. **p < 0.01

To examine the biochemical basis of the significance of Rpn13, we generated liver-specific Rpn13 knockout (Rpn13^LKO^) mice by crossing *Adrm1*-floxed mice with transgenic mice that expressed Cre recombinase in postnatal hepatocytes under the control of the albumin (Alb) promoter [30]. Rpn13^LKO^ mice were born without any developmental defects. We confirmed loss of Rpn13 protein in the 8-week-old Rpn13^LKO^ liver (Fig 2A). Loss of Rpn13 caused concurrent loss of Uch37, a deubiquitinating enzyme bound to Rpn13, consistent with our previous work [4]. In addition, we observed increases in protein levels of the CP subunits α3 (Q9R1P0) and β2 (P70195) and the RP subunits Rpn8 (A1L3B8) and Rpt6 (P62196) in the Rpn13^LKO^ liver, compared to the control liver (Fig 2A).

We next fractionated liver lysates by glycerol gradient centrifugation, followed by immunoblot analysis and measurement of peptidase activity of each fraction. The absence of Rpn13 and Uch37 did not affect the assembly of the 26S proteasome in Rpn13^LKO^ liver as shown by the normal distribution of Rpn10, α6 (Q3TS44) (CP), and Rpn8 (RP). As suggested by the results in Fig 2A, the amount of the 26S proteasome increased in Rpn13^LKO^ liver (Fig 2B). This observation was confirmed by increased peptidase activity in the 26S proteasome fractions of the Rpn13^LKO^ liver, compared with the control liver (Fig 2C). Consistent with this, mRNA levels of proteasome subunits in the Rpn13^LKO^ liver were increased (S3 Fig). These results suggest that absence of Rpn13 attenuates degradation of ubiquitinated proteins and induces a feedback increase in the expression of proteasome subunits [31–34].

To confirm this view, we employed the 26S proteasome fraction (fraction 20–23 in Fig 2C) for *in vitro* degradation of ubiquitinated cIAP1 (Q62210) protein [35]. The 26S fraction of Rpn13^LKO^ liver lysates contained a larger amount of the 26S proteasome than that of control liver lysates, and hence exhibited higher peptidase activity (Fig 2B, 2C and 2D, left panel). Therefore, the degradation rate of cIAP1 was normalized by the peptidase activity. This revealed that the 26S proteasome without Rpn13 had a moderate defect in degrading ubiquitinated cIAP (Fig 2D, right panel). Also, the 26S proteasome lacking Rpn13 showed low deubiquitinating activity compared to the control 26S proteasome, most likely due to loss of Uch37 (Fig 2E). These results indicate that Rpn13 plays an important role in degradation of ubiquitinated proteins in the mouse liver.

### Simultaneous deletion of Rpn10-UIM and Rpn13 causes severe liver injury

We previously reported that mice lacking the UIMs of Rpn10 (Rpn10^ΔUIM^) are embryonic lethal and that liver-specific ablation of Rpn10-UIM (Rpn10^LΔUIM^) caused accumulation of ubiquitinated proteins in the liver [21]. Since *Δrpn10* and *Δrpn13* leads to synthetic defects in proteasome function in *S*. *cerevisiae* [12,36], we next examined whether absence of Rpn10-UIM and Rpn13 also exhibit a synthetic effect in mouse liver.

**Fig 3:**
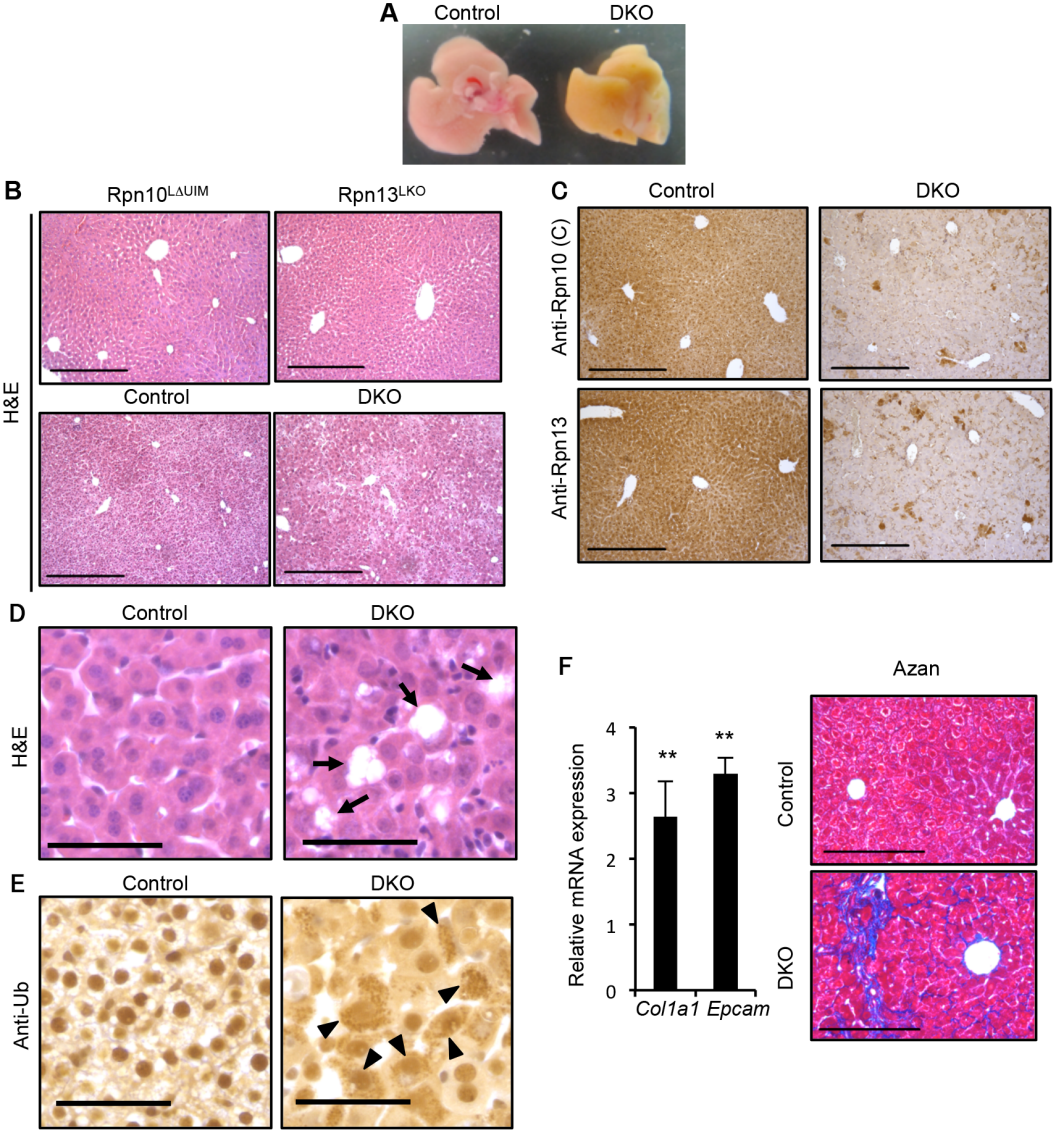
Simultaneous deletion of Rpn10-UIM and Rpn13 causes severe liver injury. (A) Representative macroscopic images of 2-week-old control and DKO livers. (B) H&E staining of 4-week-old Rpn10^LΔUIM^, Rpn13^LKO^ and 2-week-old control and DKO livers. All scale bars (black lines), 300 μm. (C) Immunohistochemical analysis on representative liver paraffin sections from 2-week-old mice by using Rpn10 (C) and Rpn13 antibodies. Scale bars, 300 μm. (D and E) Representative H&E staining (D) and immunohistochemical analysis of ubiquitin (E) on liver sections from 2-week-old mice. Arrows in (D) indicate regions of sloughing hepatocytes. Arrowheads in (E) indicate hepatocytes with high accumulation of ubiquitin in cytosol. All scale bars (black lines), 50 μm. (F) Azan staining of liver sections from 5-week-old control and DKO mice (right panels). All scale bars (black lines), 200 μm. Real-time RT-PCR was used to measure the expression of transcripts encoding fibrosis markers (*Col1a1* and *Epcam*) in the livers of 3–6-week-old control and DKO mice (left panels). Data represent levels of transcripts in each genotype liver relative to those in control liver and are expressed as means; error bars denote SEM. **p < 0.01 (n = 4 each genotype).

Liver-specific double knockout for Rpn10-UIM and Rpn13 (DKO) caused an aberrantly icteric liver at two weeks of age, compared to controls (Fig 3A). Histological analysis of these livers as well as each single knockout liver revealed that whereas either Rpn10^LΔUIM^ or Rpn13^LKO^ livers had almost normal liver histology indistinguishable from the control liver, DKO livers had disorganized architecture (Fig 3B). Immunohistochemistry using antibodies against the Rpn10 C-terminus (corresponding to the UIM domain) and Rpn13 proteins confirmed the absence of both Rpn10-UIM and Rpn13 proteins in the DKO liver (Fig 3C). The DKO liver exhibited multiple areas of hepatocyte loss (Fig 3D) and hypertrophic hepatocytes (S4 Fig). Immunostaining with an anti-ubiquitin antibody detected strong ubiquitin-positive signals in the cytosol of DKO hepatocytes (Fig 3E). We also observed vacuolated nuclei in the DKO hepatocytes (S4 Fig). At 5 weeks of age, the expression of the extracellular matrix genes *Col1a1* (12842) and *Epcam* (17075) were upregulated in DKO livers (Fig 3F, left panel), and as a consequence DKO liver displayed fibrosis as detected by Azan staining (Fig 3F, right panel); chronic hepatitis and cholestasis are known to lead to liver fibrosis [37]. At 7 weeks of age, focal necrosis was apparent in the DKO liver (S4 Fig).

Consistent with the icteric appearance of the DKO liver, serum markers of cholestasis such as alkaline phosphatase (ALP), γ-glutamyltranspeptidase (γ-GTP), cholesterol, bilirubin, and bile acid, as well as markers of liver injury (alanine and asparatate aminotransferases; ALT, AST) were significantly increased in 4-week-old DKO mice (S4 Fig), suggesting that either impairment of the biliary system or hepatocyte death is the cause of the liver pathology. We employed quantitative RT-PCR analysis to assess expression of genes involved in the biliary system. Reduction in *Slc10a1* (20493), which mediates bile acid uptake into hepatocytes, and increase in *Abcc4* (239273), which promotes efflux of bile acid from hepatocytes, suggests compensatory regulation to reduce the high concentration of bile acid in the DKO liver. This is consistent with the expression pattern observed in liver injury (S4 Fig) [38]. Furthermore, the expression of genes involved in bile acid synthesis (*Cyp8b1* [13124] and *Cyp7a1* [13122]) was not increased in DKO liver (S4 Fig). These results suggest that the principal defect resulting from simultaneous loss of Rpn10 and Rpn13 is hepatocyte injury.

### Redundant roles of Rpn10 and Rpn13 in degradation of ubiquitinated proteins

**Fig 4:**
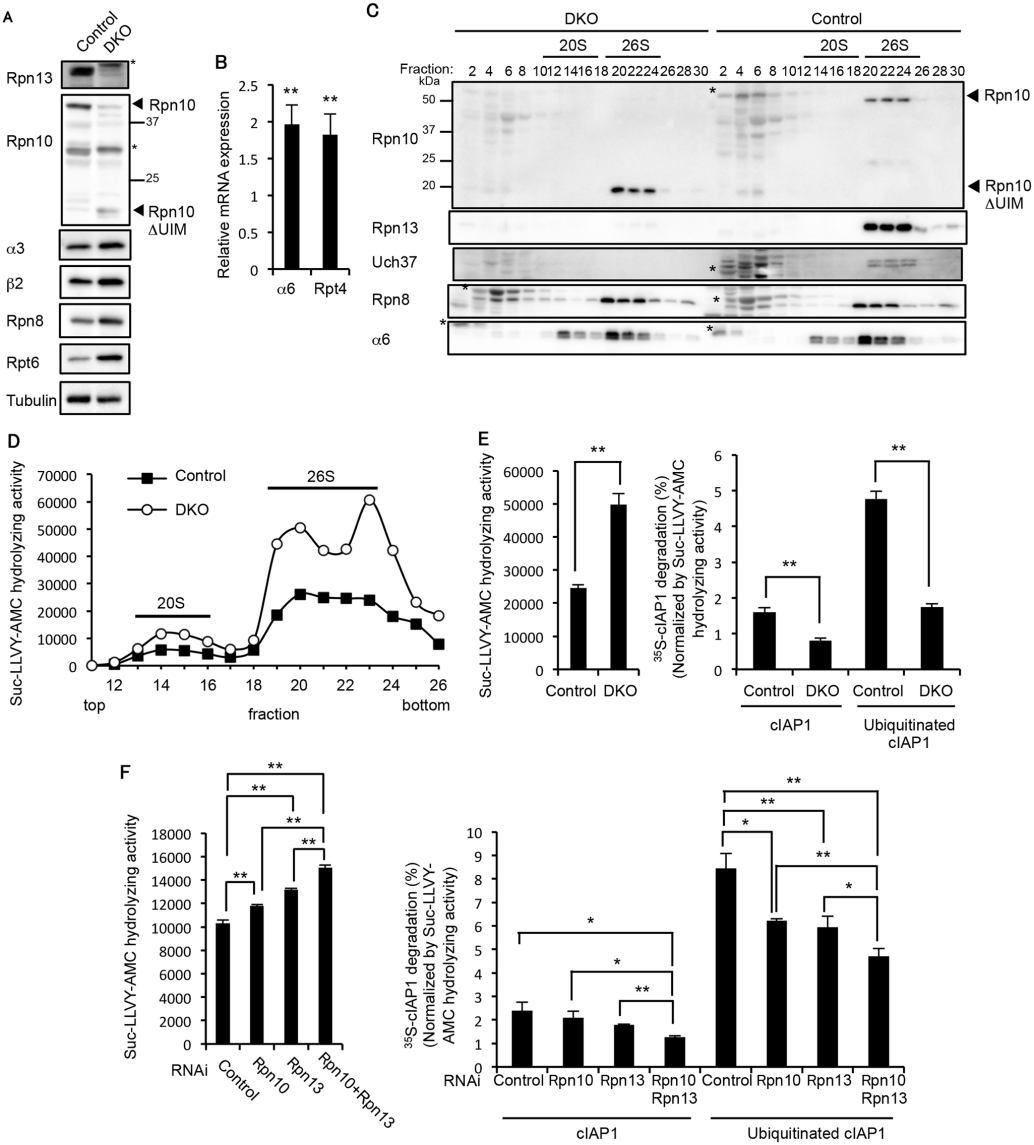
Redundant roles of Rpn10 and Rpn13 in degradation of ubiquitinated proteins. (A) Immunoblot analysis of liver lysates from 3-week-old control and DKO mice with antibodies against the indicated proteins. (B) Real-time RT-PCR was performed to measure the mRNA expressions of the proteasome subunits α6 and Rpt4 in the liver of 2–4-week-old control and DKO mice. Data represent transcript levels in DKO livers relative to those in control livers and are expressed as means; error bars denote SEM. **p < 0.01 (n = 7 for each genotype). (C) Lysates from control and DKO livers were fractionated by glycerol gradient centrifugation (8 to 32% glycerol from fraction 1 to 30) and an equal amount of each fraction was used for immunoblot analysis using antibodies against the indicated proteins. Asterisks indicate nonspecific bands. (D) Each fraction of (C) was assayed for chymotrypsin-like activity using Suc-LLVY-AMC as a substrate. (E) The 26S proteasome fractions of (D) (fractions 20–23) were subjected to the assay of chymotrypsin-like activity (left panel). Degradation rates of ^35^S-labeled cIAP1 with or without ubiquitination were measured and normalized by chymotrypsin-like activity (right panel). Data are mean ± standard deviations from triplicate experiments. **p < 0.01. (F) Lysates from HeLa cells transfected with indicated siRNAs were assayed for Suc-LLVY-AMC hydrolyzing activity (left panel) and degradation of ^35^S-labeled cIAP1 with or without ubiquitination. cIAP degradation rates are normalized by Suc-LLVY-AMC hydrolyzing activity (right panel). Data are mean ± standard deviations from three experiments. *p < 0.05; **p < 0.01.

We next examined how deficiency of Rpn10-UIM and Rpn13 affects proteasome function. We confirmed the loss of both the full-length Rpn10 and Rpn13 proteins in the 3-week-old DKO liver (Fig 4A). In addition, increases in the CP and RP subunits were observed in the DKO liver, suggesting that expression of proteasome subunits was induced in the DKO liver by the feedback mechanism (Fig 4A). Indeed, we confirmed increased mRNA expression of CP and RP subunits in the DKO liver (Fig 4B). The assembly of the 26S proteasome was normal in DKO livers, as revealed by normal distribution of the CP subunit α6 and the RP subunit Rpn8 (Fig 4C). The truncated form of Rpn10 (Rpn10ΔUIM) was incorporated correctly into 26S proteasomes, as we previously reported [21]. Loss of Uch37 proteins was also observed in DKO livers (Fig 4C). Consistent with the increase in assembled proteasomes, the proteasome peptidase activity of the DKO liver was nearly twice than that of the control liver (Fig 4D).

To assess the ubiquitin-mediated protein degrading activity of DKO proteasomes, the 26S proteasome fractions (fraction 20–23 in Fig 4D) were subjected to an *in vitro* degradation assay of ubiquitinated cIAP1, and activity was normalized by the respective peptidase activities (Fig 4E, left panel). The degradation rate of ubiquitinated cIAP1 was markedly reduced in the DKO proteasome compared to the control proteasome (Fig 4E, right panel), suggesting severe defects in degradation of ubiquitinated proteins in the absence of both Rpn10 and Rpn13. Intriguingly, degradation of unmodified cIAP1 was also reduced in the DKO proteasome compared to the control proteasome (Fig 4E, right panel). Although the mechanism of ubiquitin-independent protein degradation by the 26S proteasome is not fully understood, some structural changes caused by loss of Rpn13 and the UIM domain of Rpn10 might affect the degradation mechanism. Similar results were obtained using lysates of HeLa cells treated with siRNAs against Rpn10, Rpn13, or combinations of these. Rpn10/Rpn13-double knockdown caused significant increase in peptidase activity compared to single knockdown of either Rpn10 or Rpn13 (Fig 4F, left panel). The degradation rate of ubiquitinated cIAP1 was significantly reduced in Rpn10/Rpn13-double knockdown compared to single knockdown of either Rpn10 or Rpn13 (Fig 4F, right panel). These results indicate that the Rpn10 loss and Rpn13 loss synthetically affect degradation of ubiquitinated proteins.

### Defective binding of ubiquitinated proteins and the UBL-UBA proteins mHR23B and ubiquilins to Rpn10ΔUIM/ΔRpn13 proteasomes

**Fig 5:**
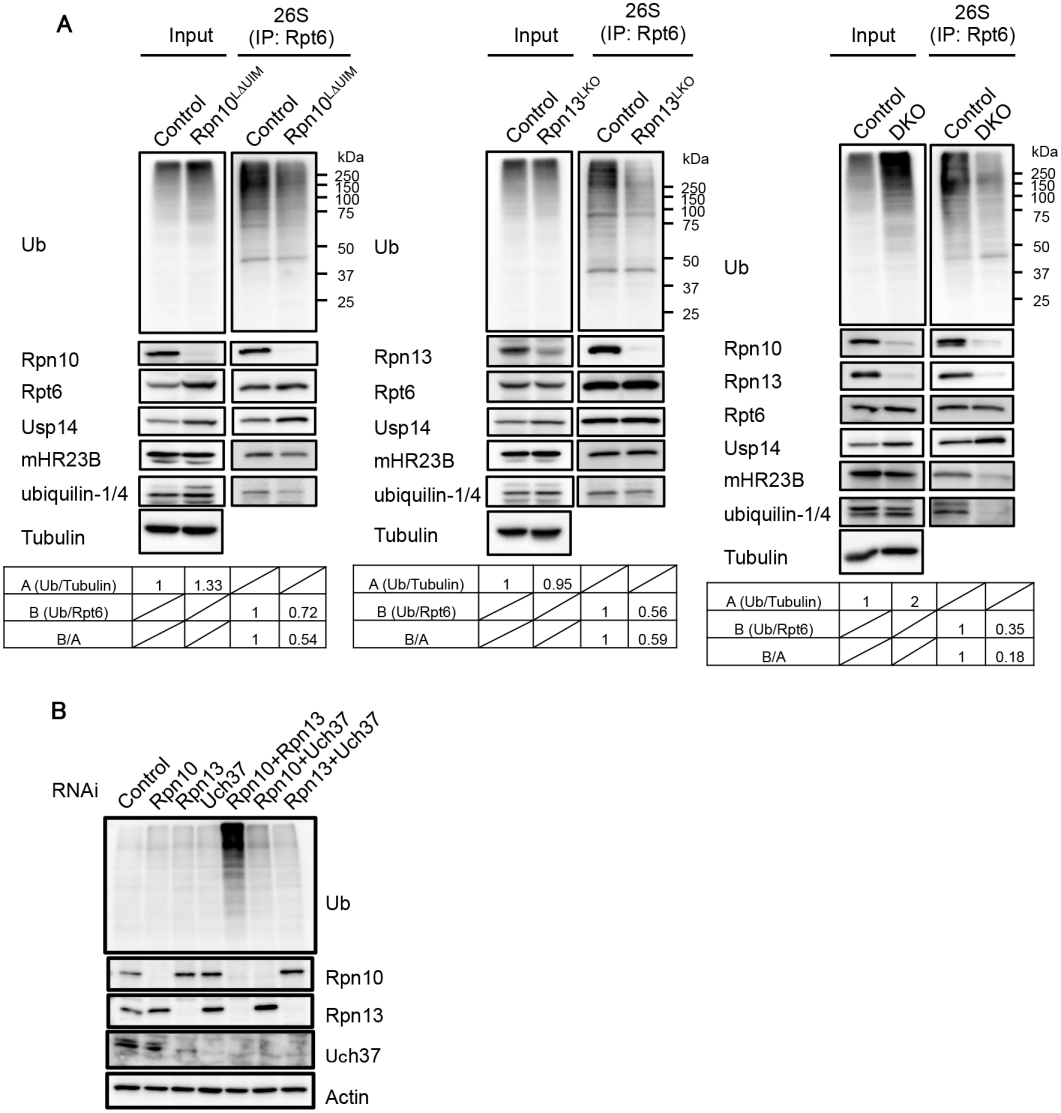
Defective binding of ubiquitinated and UBL-UBA proteins to Rpn10ΔUIM/ΔRpn13 proteasomes. (A) Homogenates from mouse livers were immunoprecipitated with an anti-Rpt6 antibody and subjected to immunoblotting with the indicated antibodies. Values for the relative band intensities of ubiquitin normalized by tubulin (input) or Rpt6 (IP) are shown as A and B, with the control being set to one. Values for B/A indicate the relative amount of bound ubiquitinated proteins to the amount of input ubiquitinated proteins. (B) HEK293T cells were transfected with siRNA against Rpn10, Rpn13, or Uch37. Where indicated, cells were transfected with a mixture of siRNAs. After 96h, cell extracts were subjected to SDS-PAGE, followed by immunoblotting with the indicated antibodies.

Since both Rpn10-UIM and Rpn13 can recognize ubiquitin chains, we examined whether binding of ubiquitinated proteins to the 26S proteasome was affected in the Rpn10^LΔUIM^, Rpn13^LKO^, and DKO livers. The 26S proteasome was immunoprecipitated from each liver lysate and subjected to immunoblot analysis. While the amount of ubiquitinated proteins was increased in Rpn10^LΔUIM^, the amount of ubiquitinated proteins coprecipitated with the 26S proteasome was decreased in Rpn10^LΔUIM^ livers compared to control (Fig 5A, left panel). The amount of ubiquitinated proteins in Rpn13^LKO^ livers was similar to that in control livers, but ubiquitinated proteins that coprecipitated with the 26S proteasome was decreased in Rpn13^LKO^ livers compared to control (Fig 5A, middle panel). These results indicate that either loss of Rpn10 or Rpn13 causes failure in recruiting ubiquitinated proteins to the proteasome.

In DKO liver, the accumulation of ubiquitinated proteins was augmented compared to each single knockout liver, yet the amount of co-precipitated ubiquitinated proteins was much smaller than the control liver, especially in terms of the relative amount of bound ubiquitinated proteins to that of input ubiquitinated proteins (Fig 5A, right panel). We also observed an increase in Usp14 bound to the Rpn10^LΔUIM^ and DKO proteasomes (Fig 5A, left and right panels), consistent with previous observations that ubiquitin stress enhances binding of Ubp6 (P43593), a Usp14 ortholog, with the proteasome [7,21,39]. The reduction in proteasome-bound ubiquitinated proteins strongly suggests that recruitment of ubiquitinated proteins is severely impaired in proteasomes lacking Rpn10-UIM or Rpn13 and that simultaneous deletion of Rpn10-UIM and Rpn13 enhances this effect.

Ubiquitinated proteins are either directly recognized by Rpn10 and Rpn13 or bound and delivered to the proteasome by UBL-UBA proteins [14]. To examine whether the association of UBL-UBA proteins with the proteasome was affected in the absence of Rpn10-UIM and Rpn13, the immunoprecipitates were also probed for mHR23B and ubiquilin-1/4 (the antibody detects both proteins). mHR23B bound to Rpn10^LΔUIM^ proteasomes was reduced compared to that bound to the control proteasomes (Fig 5A, left panel), consistent with our previous observations [21]. The amount of ubiquilin-1/4 bound to Rpn10^LΔUIM^ proteasomes was also decreased (Fig 5A, left panel). On the other hand, the amount of mHR23B and ubiquilin-1/4 in Rpn13^LKO^ proteasomes were comparable to and slightly decreased compared to control proteasomes, respectively (Fig 5A, middle panel). However, mHR23B and ubiquilin-1/4 were only faintly detected in DKO proteasomes (Fig 5A, right panel). To date, Rpn1 has been considered the major subunit for recruitment of UBL-UBA proteins in *S*. *cerevisiae* [40–42]. These results demonstrate that both Rpn10 and Rpn13 are the major subunits for recruitment of mHR23B and ubiquilin-1/4 in mammals, where Rpn10 and Rpn13 play partially redundant roles. These results also suggest that accumulation of ubiquitinated proteins is due not only to failure in direct recognition of ubiquitin chains by Rpn10 and Rpn13, but also impairment in delivery of ubiquitinated proteins by mHR23B and ubiquilin-1/4.

Since deletion of Rpn13 leads to concurrent loss of Uch37, the possibility remains that loss of Uch37 rather than Rpn13 is responsible for the massive accumulation of ubiquitinated proteins in the absence of Rpn13. To exclude this possibility, we compared the levels of ubiquitinated proteins in HEK293T cells treated with siRNAs against Rpn10, Rpn13, Uch37, or combinations of these (Fig 5B). Consistent with the results in mice, Rpn10/Rpn13-double knockdown caused profound accumulation of ubiquitinated proteins compared to single knockdowns of either Rpn10 or Rpn13. Notably, Rpn10/Uch37-double knockdown did not cause such accumulation (Fig 5B), indicating that the synthetic effect of Rpn13 deletion with Rpn10 deletion is not due to loss of Uch37 but due to loss of Rpn13 itself. These results also show that the effects of Rpn10 loss and Rpn13 loss work synthetically. Taken together with the histological analysis (Fig 3), these results suggest that a large part of ubiquitinated proteins can be recognized either through Rpn10 or Rpn13, whether directly or indirectly, and that the roles of Rpn10 and Rpn13 are largely, if not entirely, redundant.

### Synthetic effect of Rpn10 and Rpn13 deletions on cellular stress

**Fig 6:**
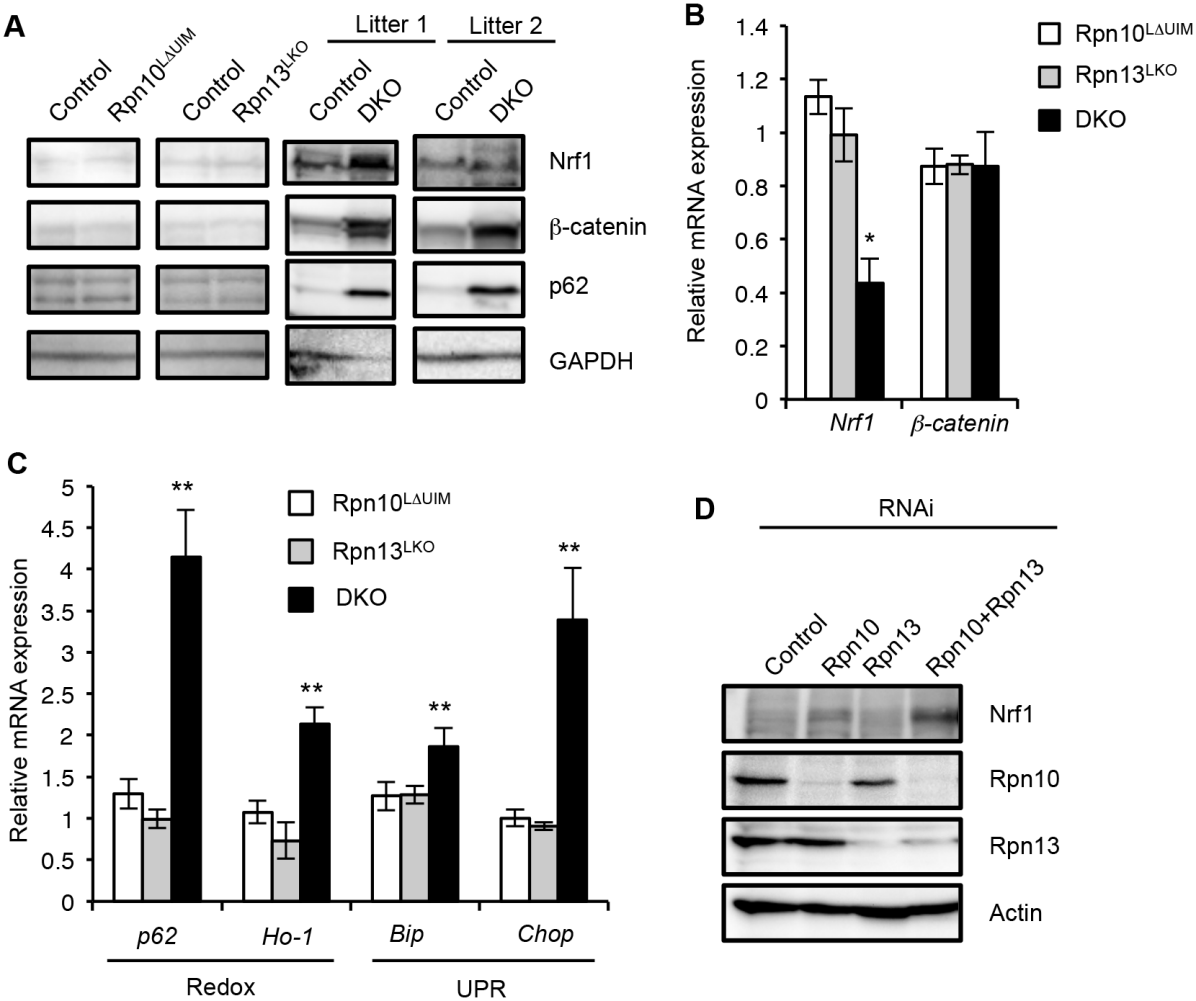
Synthetic effect of Rpn10-UIM and Rpn13 deletion on degradation of ubiquitinated proteins and cellular stress. (A) Immunoblot analysis of whole-cell extracts of livers from indicated genotypes of mice (2-month-old for Rpn10^LΔUIM^ and Rpn13^LKO^, and 2–4-week-old DKO) with antibodies against indicated proteins. (B and C) Real-time RT-PCR was used to measure the expression of transcripts encoding *Nrf1*, *β-catenin*, redox pathway (*p62* [18412] and *Ho-1* [15368]), and UPR pathway (*Bip* [14828] and *Chop* [13198]) genes in the livers of 3–6-week-old control and DKO mice. Data represent levels of transcripts in each genotype liver relative to those in control liver and are expressed as means; error bars denote SEM. *p < 0.05; **p < 0.01 (n = 3 for Rpn10^LΔUIM^ and Rpn13^LKO^, and n = 4 for DKO). (D) HeLa cells were transfected with siRNA against Rpn10 and Rpn13. Where indicated, cells were transfected with a mixture of siRNAs. After 96h, whole-cell extracts were subjected to SDS-PAGE, followed by immunoblotting with the indicated antibodies.

To further test redundant roles of Rpn10-UIM and Rpn13 in degradation of ubiquitinated proteins in the liver, we examined the protein levels of Nrf1 (Q61985) and β-catenin (Q02248), both of which are constitutively ubiquitinated and constantly degraded by the proteasome [34,43]. The protein levels of Nrf1 and β-catenin in Rpn10^LΔUIM^ and Rpn13^LKO^ single knockout livers were comparable to those in the control liver. However, both proteins were remarkably accumulated in the DKO liver, although the extent varied between litters (Fig 6A and S5 Fig). These increases are likely due to a defect in protein turnover, because mRNAs of *Nrf1* (*Nfe2l1*) (18023) and *β-catenin* (*Ctnnb1*) (12387) were not increased in the DKO liver (Fig 6B). These results support the view that Rpn10 and Rpn13 act redundantly to degrade ubiquitinated proteins in general.

It has been reported that inhibition of the proteasome causes oxidative stress and ER stress, which then induce expressions of redox genes and the unfolded protein response (UPR), respectively [44,45]. We therefore examined whether induction of such genes is also exaggerated when both Rpn10-UIM and Rpn13 are deficient. Quantitative RT-PCR analysis showed a remarkable increase in mRNA for redox genes (*p62* and *Ho-1*) and UPR genes (*Bip* and *Chop*) in DKO livers, compared to the single knockout mice (Fig 6C). The increase in p62 proteins is likely due to upregulation of *p62* expression (Fig 6A). We also confirmed accumulation of Nrf1 proteins in Rpn10/Rpn13-double knockdown HeLa cells (Fig 6D). These results indicate that simultaneous loss of Rpn10-UIM and Rpn13 induces cellular stress more severe than loss of either Rpn10-UIM or Rpn13 and further supports the view that Rpn10 and Rpn13 act redundantly for cellular homeostasis.

### Liver injury in DKO liver induce regeneration accompanied by fibrosis

**Fig 7:**
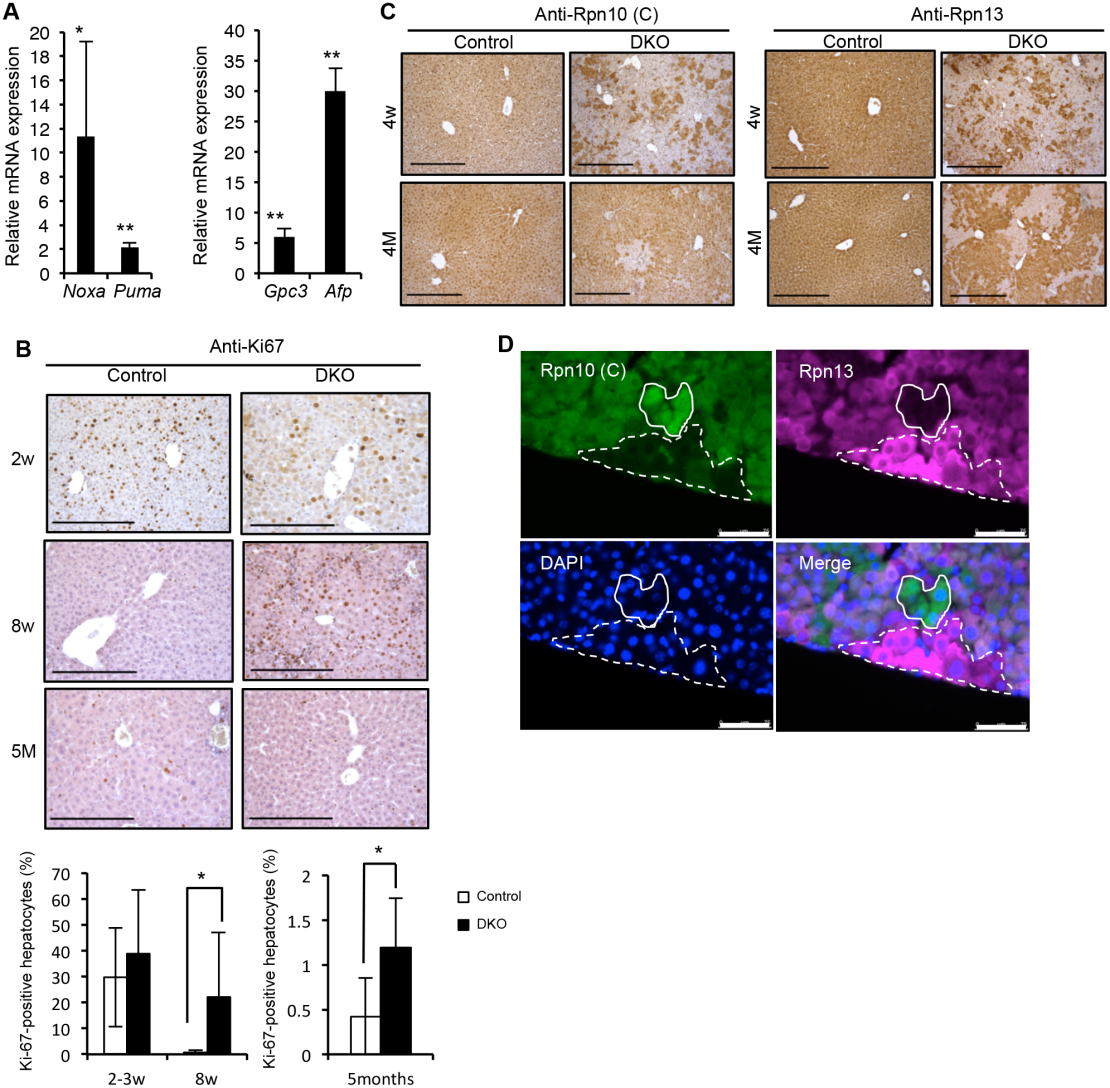
Spontaneous liver injury, fibrosis and regeneration in DKO mice. (A) Real-time RT-PCR was used to measure the expressions of p53 target pro-apoptotic related genes *Noxa* and *Puma* (left panel) and proliferation marker genes *Gpc3* and *Afp* (right panel) in the livers of 3–6-week-old control and DKO mice. Data represent levels of transcripts in each genotype liver relative to those in control liver and are expressed as means; error bars denote SEM. *p < 0.05; **p < 0.01 (n = 4 each genotype). (B) Representative liver sections of 2- (top panels), 8-week (middle panels), and 5-month-old (bottom panels) control and DKO mice were stained for Ki-67 followed by quantification of the percentage of Ki-67 positive proliferating hepatocytes calculated from three high-power-fields (HPF) analysis. Results are shown as means, and error bars indicate SEM (n = 3–7 mice for each genotype). All scale bars (black lines), 100 μm. (C) Immunohistochemical analyses on representative liver paraffin sections from 4-week-old and 4-month-old mice by using Rpn10 C-terminal antibodies, refer to Rpn10 (C), and Rpn13 antibodies. All scale bars (black lines), 300 μm. (D) Immunofluorescent analysis of liver sections from 4-week-old DKO mice by using Rpn10 (C) and Rpn13 antibodies. 4’, 6-Diamidino-2-phenylindole (DAPI) was used for nuclear counterstaining. The dashed line region represents Rpn13 (+)/Rpn10 (-) hepatocytes, while the lined region represents Rpn13 (-)/Rpn10 (+) hepatocytes. All scale bars (white lines), 75 μm.

Despite severe liver injury at 2 weeks of age (Fig 3A–3E), DKO mice survived as long as 50 weeks. We examined the expression of pro-apoptotic genes (*Noxa* [58801] and *Puma* [170770]) and proliferation marker genes (*Gpc3* [14734] and *Afp* [11576]) in 3–6-week-old mice (Fig 7A). This analysis revealed that both pro-apoptotic and proliferation-related genes were upregulated in the DKO liver. Indeed, immunostaining of liver sections for the proliferation marker Ki-67 confirmed hepatocyte proliferation in DKO mice, especially at 8 weeks of age (Fig 7B). These results suggest that liver regeneration following loss of hepatic tissue caused by liver injury occurs in DKO mice.

We then immunostained the liver sections for the expression of Rpn10-UIM and Rpn13. At 4 weeks of age, the majority of hepatocytes expressed neither Rpn10-UIM nor Rpn13, but compared to 2 weeks of age, some Rpn10-UIM- or Rpn13-positive areas appeared (Figs 3C and 7C). At 4 months of age, the majority of hepatocytes were Rpn10-UIM- or Rpn13-positive (Fig 7C). These results demonstrate that liver regeneration by hepatocytes expressing either Rpn10 or Rpn13 occurred in adult DKO liver.

We observed clusters of cells strongly expressing either Rpn10 or Rpn13 in DKO liver, although the mechanism by which these cells recovered expression of Rpn10 and Rpn13 is unclear (Fig 7D). These results indicate that recovery of either Rpn10 or Rpn13 is sufficient to regenerate hepatocytes, further demonstrating that hepatocytes lacking both Rpn10 and Rpn13 are unable to survive and proliferate in mammals.

## Discussion

In this study, we showed that Rpn13 plays a redundant role with Rpn10 in recognition and degradation of ubiquitinated proteins in mouse livers. Hepatocyte-specific ablation of either Rpn10-UIM or Rpn13 did not cause obvious defects and showed only mild accumulation of ubiquitinated proteins. However, simultaneous loss of both Rpn13 and Rpn10-UIM caused failure in degradation of ubiquitinated proteins both *in vivo* and *in vitro* and led to intense accumulation of ubiquitintead proteins in hepatocytes. An NMR study showed that Rpn10 and Rpn13 can bind simultaneously to a single diubiquitin, where Rpn13 and Rpn10 preferably bind to the proximal and distal ubiquitin, respectively [20]. In addition, proteasome structures obtained by electron microscopy showed that the distance between Rpn10 and Rpn13 is long enough to determine the minimal length for a ubiquitin chain to be recognized by the proteasome, and that simultaneous binding of Rpn10 and Rpn13 to a single ubiquitin chain can orient the chain for deubiquitination, which might promote degradation of ubiquitinated proteins [46,47]. However, our results suggest that most proteins are efficiently degraded when either Rpn10 or Rpn13 is intact, as shown by accumulation of ubiquitinated proteins (Fig 5A and 5B), accumulation of Nrf1 and β-catenin (Fig 6A and 6D), and liver injury (Figs 3 and 6C). On the other hand, some proteins accumulated in the absence of Rpn10-UIM or Rpn13 could be specific substrates for either Rpn10 or Rpn13. Considering that both Rpn10- and Rpn13-deficient mice are embryonic lethal, there are also non-redundant roles for these receptors, and defects in degradation of each receptor-specific substrates might be the cause of lethality in Rpn10- and Rpn13-deficient mice. The specificity may be determined by the relative position and orientation between the ubiquitinated site and the degradation initiation site of the substrate [48].

An unexpected finding is that the UBL-UBA proteins mHR23B and ubiquilin-1 and -4 were only faintly detected in 26S proteasomes lacking both Rpn10-UIM and Rpn13 (Fig 5A). In *S*. *cerevisiae*, Rpn1 has been shown to contribute to recruitment of UBL-UBA proteins [14,40]. However, our results clearly indicate that Rpn10 and Rpn13 are major acceptors of mHR23B and ubiquilin-1 and -4 in mice. *S*. *cerevisiae* Rpn10 has only one UIM, whereas mammalian Rpn10 has two UIMs. It has been reported that the first UIM binds to ubiquitin chains while the second UIM binds to the UBL domain of UBL-UBA proteins in mammalian Rpn10 [49]. It is possible that the second UIM of Rpn10, which is found in metazoa [47], has taken over the role of Rpn1 as an acceptor of UBL-UBA proteins. These results also suggest that recognition of ubiquitinated proteins by the mammalian proteasome is not the same as that by the *S*. *cerevisiae* proteasome.

Despite lack of the two ubiquitin receptor subunits and binding of mHR23B and ubiquilin-1/4, DKO proteasomes still bound ubiquitinated proteins (Fig 5A). This suggests the presence of other ubiquitin receptor(s) on the 26S proteasome. The existence of such additional ubiquitin receptor(s) had been proposed in *S*. *cerevisiae* [12,36]. Indeed, Dss1/Sem1/Rpn15 (O14140) were recently identified as additional ubiquitin receptor subunits of the proteasome in *Saccharomyces pombe* [50].

Rpn13 was found to be present in a substoichiometric amount in the proteasomes of *S*. *cerevisiae*, *D*. *melanobaster*, and human [51–53]. A recent study demonstrated that the 26S proteasome contains only one Rpn13, which defines the asymmetry of the 26S proteasome [54]. In addition, ubiquitination of Rpn13 decreases proteasome ability to bind and degrade ubiquitinated proteins [55]. Considering that the phenotype of Rpn13 mice is less severe that of Rpn10ΔUIM mice, which are lethal at embryonic day 8–9 [21], contribution of Rpn13 in degradation of ubiquitinated proteins seems to be smaller than Rpn10. Therefore, we suggest that Rpn13 plays an auxiliary regulatory role in protein degradation by the proteasome under certain conditions such as proteotoxic stress and cell proliferation, especially in tumor cells. Consistent with this, an Rpn13 inhibitor was recently reported to be toxic to tumor cells but tolerated by normal cells [56]. It has been suggested that Rpn10, Rpn13, and other ubiquitin receptor(s) may have distinct substrate specificity to cover a broad range of ubiquitinated substrates [57]. Thus, further analysis is required to reveal the complicated mechanisms of ubiquitin recognition and degradation by the 26S proteasome, which would provide useful information for elucidating physiological functions of each ubiquitin receptor and for designing drugs targeting these receptors.

## Materials and Methods

### Gene targeting of *Adrm1* (*Rpn13)*

A targeting vector for *Adrm1* (*Rpn13*) conditional deletion was constructed by inserting *loxP* sequences into intron 2 and intron 4 so that exon 3 and 4 was deleted by the expression of Cre recombinase. A neomycin cassette flanked by *FRT* sites was inserted into intron 2. RENKA embryonic stem cells were screened as described previously [58]. For Southern blot analysis, genomic DNA was digested with *Bam*HI and was hybridized with the probes shown in S1 Fig. EIIa-Cre and Alb-Cre were purchased from the Jackson Laboratory. PCR primers used for mouse genotyping are listed in S1 Table. All animal experiments were performed under the guidelines of Science Council of Japan, with approval of procedures by the Institutional Animal Care Committee of Graduate School of Pharmaceutical Sciences, the University of Tokyo (approval number M25-19). Mice were housed in pathogen-free facilities.

### Cesarean delivery

Cesarean section was performed at 19 days postcoitus, and pups were placed in a humidified thermostat-controlled chamber.

### Histological examination

Tissues were dissected, fixed in 4% paraformaldehyde, embedded in paraffin, and sectioned. Sections were stained with Mayer’s hematoxylin, followed by eosin staining. For immunohistochemical analysis, all tissue sections were subjected to antigen retrieval using the microwave method in 0.01 M citrate buffer for 10 min. After blocking, sections were incubated with primary antibodies overnight at 4°C. Sections were incubated with biotinylated secondary antibodies that were detected using VECTASTAIN ABC kits (Vector) and the DAB substrate (Sigma). For immunofluorescent analysis, sections were blocked in 0.5% goat serum in TBST and then incubated with primary antibodies, followed by Alexa 488 or 633-labeled secondary antibody (Life Technologies). Paraffin-embedded tissue sections were used for AZAN staining. Staining of skeleton was performed with Alizarin red and Alcian blue. Serum tests were performed by a commercial laboratory (Oriental Yeast Co., Ltd.).

### RNA isolation, reverse transcription, and real-time PCR

For real-time PCR analysis, total RNAs were isolated from the livers of 5-week-old mice by using an RNAspin kit (Roche), reverse transcribed to cDNA using a SuperScript VILO cDNA Synthesis Kit (Life Technologies), and subjected to real-time PCR using the Light cycler 480 system (Roche). PCR primers and universal probes (Roche), which are listed in S2 Table, were designed according to the Universal Probe Assay Design Center. *Glucuronidase beta* (*Gusβ*) was used for normalization. Real-time PCR data were analyzed by the ΔΔC_*T*_ method.

### Immunological analysis

Mouse livers were homogenized and subjects to immunoblotting and immunoprecipitation as described previously [21]. Whole-cell extracts from the livers and HeLa cells were homogenized and lysed in buffer containing 50 mM Tris-HCl (pH 7.5), 1% SDS, 5 mM EDTA, and 10 mM 2-ME, followed by sonication. The antibodies for α3, α6, β2, Rpt6, Rpn8, Rpn10 (N), Rpn10 (C), Rpn13, Uch37, mHR23B, Usp14, and polyubiquitin were described previously [21, 59]. A polyclonal antibody that recognizes both ubiquilin-1 and ubiquilin-4 was raised by immunizing rabbits with recombinant full-length ubiquilin-4 proteins. For immunohistochemistry, monoclonal antibodies against Rpn13 were raised by immunizing rats with recombinant full-length Rpn13 proteins. The antibodies for Tubulin (sc-5286; Santa Cruz Biotechnology), Ki-67 (RM-9106; Thermo Scientific), Nrf1 (sc-13031; Santa Cruz Biotechnology), β-catenin (610153; BD biosciences), p62 (PM045; MBL), Actin (MAB1501R; Millipore), and GAPDH (MCA4739; AbD Serotec) were purchased. For immunoprecipitation, liver homogenates were immunoprecipitated with an anti-Rpt6 antibody as described previously [21]. Band intensities were quantified using Fusion software (M&S Instruments Inc.).

### Glycerol gradient analysis

Liver homogenates were clarified by centrifugation at 20,000 x g and subjected to 8 to 32% (vol/vol) linear glycerol gradient centrifugation (22 h, 83,000 x g) as described previously [59].

### Assay of proteasome activity

The assays of proteasome chymotryptic peptidase activity, deubiquitination assay, and degradation of polyubiquitinated ^35^S-labeled cIAP1 protein have been described previously [4].

### RNAi experiments

siRNA-mediated knockdown was performed as described previously. The targeted sequences are as follows: Rpn10, 5’-GGAGCAGAGUUUGGCCAGGCGGAAU-3’; Rpn13, 5’-GGAGGGUCUACGUGCUGAAGUUCAAA-3’; Uch37, 5’-ACCGAGCTCATTAAAGGATTCGGTT-3’. Cells were harvested 96 h after transfection of siRNAs [4].

## Supporting Information

S1 Fig Gene targeting of *Adrm1*. (A) Schematic representation of the targeting vector and the targeted allele of the *Adrm1* (*Rpn13*) gene. Exons 1 to 9 are shown as solid rectangles. The probe for Southern blot analysis is shown as a gray box. The positions of PCR primers are depicted as arrows. Neo, neomycin-resistant cassette; DTA, diphtheria toxin gene. (B) Southern blot analysis of genomic DNAs extracted from mouse tails. WT and Flox alleles were detected as 14-kb and 8-kb bands, respectively.
(DOCX)

S2 Fig Rpn13KO shows neonatal lethality. (A) Gross appearance (upper panels) of placenta from control and Rpn13KO E18.5 embryos. H&E stained sections (bottom panels) of placenta from control and Rpn13KO E15.5 embryos. (B) Gross appearance (upper panels) and H&E stained sections (bottom panels) of heart from control and Rpn13KO neonates. (C) Lung sections from littermate control and Rpn13KO embryos were prepared at the indicated stages and stained with H&E.
(DOCX)

S3 Fig Rpn13 deficiency in the liver induces the expression of proteasome subunits. Real-time RT-PCR was performed to measure the expressions of proteasome subunits such as *α6* (*Psma1*), *Rpn6* (*Psmd11*), and *Rpt4* (*Psmc6*) in the liver of 6-week-old control and Rpn13^LKO^ mice. Data represent levels of transcripts in each genotype liver relative to those in control liver and are expressed as means; error bars denote SEM.
(DOCX)

S4 Fig Aberrant morphology of hepatocytes and injury in DKO liver. (A and B) H&E stained section (A) and immunohistochemical analysis (B) on representative liver paraffin sections from 2-week-old mice. Scale bars, 50 μm. (C) H&E stained sections of liver from 7-week-old control and DKO. Scale bars, 200 μm. (D) Serum level analysis of asparate aminotransferase (AST), alanine aminotransferase (ALT), alkaline phosphatase (ALP), γ-glutamyltranspeptidase (GTP), total cholesterol, total bilirubin and total bile acid in 3–6-week-old mice. Results are shown as mean ± SEM. *p < 0.05; **p < 0.01 (n = 4 each genotype). (E) Real-time RT-PCR was performed to measure the expression of transcripts encoding bile acid synthesis and transport pathways in the livers of 3–6-week-old control and DKO mice. Genes involved in bile acid efflux and uptake into hepatocytes (*Slc10a1* and *Abcc4*) and synthesis of neutral and hydrophobic bile acid (*Cyp8b1* and *Cyp7a1*) were measured. Data represent levels of transcripts in each genotype liver relative to those in control liver and are expressed as means; error bars denote SEM. *p < 0.05; **p < 0.01 (n = 4 each genotype).
(DOCX)

S5 Fig Synthetic effect between Rpn10-UIM and Rpn13 on ubiquitin mediated protein degradation. Immunoblot analysis of whole-cell extracts of livers from 2–4-week-old control and DKO with antibodies against indicated proteins.
(DOCX)

S1 Table PCR primers for genotyping. Primers a, b, and c were used for Rpn13 flox mice genotyping. Primers a and d were used for Rpn13 knockout mice genotyping.
(DOCX)

S2 Table PCR primers and universal probes for real time PCR. (DOCX)
