# Supplementary material for: Redundant Roles of Rpn10 and Rpn13 in Recognition of Ubiquitinated Proteins and Cellular Homeostasis
Source: PLoS Genet. 2015 Jul 29;11(7):e1005401. doi: 10.1371/journal.pgen.1005401 (PMC4519129; doi:10.1371/journal.pgen.1005401)
Supplement: S1 Fig — (A) Schematic representation of the targeting vector and the targeted allele of the Adrm1 (Rpn13) gene. Exons 1 to 9 are shown as solid rectangles. The probe for Southern blot analysis is shown as a gray box. The positions of PCR primers are depicted as arrows. Neo, neomycin-resistant cassette; DTA, diphtheria toxin gene. (B) Southern blot analysis of genomic DNAs extracted from mouse tails. WT and Flox alleles were detected as 14-kb and 8-kb bands, respectively. (DOCX) [file pgen.1005401.s001.docx]

**S1 Fig. Gene targeting of *Adrm1*.**

(A) Schematic representation of the targeting vector and the targeted allele of the *Adrm1* (*Rpn13*) gene. Exons 1 to 9 are shown as solid rectangles. The probe for Southern blot analysis is shown as a gray box. The positions of PCR primers are depicted as arrows. Neo, neomycin-resistant cassette; DTA, diphtheria toxin gene. (B) Southern blot analysis of genomic DNAs extracted from mouse tails. WT and Flox alleles were detected as 14-kb and 8-kb bands, respectively.
