# Supplementary material for: Redundant Roles of Rpn10 and Rpn13 in Recognition of Ubiquitinated Proteins and Cellular Homeostasis
Source: PLoS Genet. 2015 Jul 29;11(7):e1005401. doi: 10.1371/journal.pgen.1005401 (PMC4519129; doi:10.1371/journal.pgen.1005401)
Supplement: S2 Fig — (A) Gross appearance (upper panels) of placenta from control and Rpn13KO E18.5 embryos. H&E stained sections (bottom panels) of placenta from control and Rpn13KO E15.5 embryos. (B) Gross appearance (upper panels) and H&E stained sections (bottom panels) of heart from control and Rpn13KO neonates. (C) Lung sections from littermate control and Rpn13KO embryos were prepared at the indicated stages and stained with H&E. (DOCX) [file pgen.1005401.s002.docx]

**S2 Fig. Rpn13KO shows neonatal lethality.**

(A) Gross appearance (upper panels) of placenta from control and Rpn13KO E18.5 embryos. H&E stained sections (bottom panels) of placenta from control and Rpn13KO E15.5 embryos. (B) Gross appearance (upper panels) and H&E stained sections (bottom panels) of heart from control and Rpn13KO neonates. (C) Lung sections from littermate control and Rpn13KO embryos were prepared at the indicated stages and stained with H&E.
