# Supplementary material for: Redundant Roles of Rpn10 and Rpn13 in Recognition of Ubiquitinated Proteins and Cellular Homeostasis
Source: PLoS Genet. 2015 Jul 29;11(7):e1005401. doi: 10.1371/journal.pgen.1005401 (PMC4519129; doi:10.1371/journal.pgen.1005401)
Supplement: S3 Fig — Real-time RT-PCR was performed to measure the expressions of proteasome subunits such as α6 (Psma1), Rpn6 (Psmd11), and Rpt4 (Psmc6) in the liver of 6-week-old control and Rpn13LKO mice. Data represent levels of transcripts in each genotype liver relative to those in control liver and are expressed as means; error bars denote SEM. (DOCX) [file pgen.1005401.s003.docx]

**S3 Fig. Rpn13 deficiency in the liver induces the expression of proteasome subunits.**

Real-time RT-PCR was performed to measure the expressions of the proteasome subunits *α6* (*Psma1*), *Rpn6* (*Psmd11*), and *Rpt4* (*Psmc6*) in the liver of 6-week-old control and Rpn13^LKO^ mice. Data represent levels of transcripts in each genotype liver relative to those in control liver and are expressed as means; error bars denote SEM.
