# Supplementary material for: Redundant Roles of Rpn10 and Rpn13 in Recognition of Ubiquitinated Proteins and Cellular Homeostasis
Source: PLoS Genet. 2015 Jul 29;11(7):e1005401. doi: 10.1371/journal.pgen.1005401 (PMC4519129; doi:10.1371/journal.pgen.1005401)
Supplement: S4 Fig — (A and B) H&E stained section (A) and immunohistochemical analysis (B) on representative liver paraffin sections from 2-week-old mice. Scale bars, 50 μm. (C) H&E stained sections of liver from 7-week-old control and DKO. Scale bars, 200 μm. (D) Serum level analysis of asparate aminotransferase (AST), alanine aminotransferase (ALT), alkaline phosphatase (ALP), γ-glutamyltranspeptidase (GTP), total cholesterol, total bilirubin and total bile acid in 3–6-week-old mice. Results are shown as mean ± SEM. *p < 0.05; **p < 0.01 (n = 4 each genotype). (E) Real-time RT-PCR was performed to measure the expression of transcripts encoding bile acid synthesis and transport pathways in the livers of 3–6-week-old control and DKO mice. Genes involved in bile acid efflux and uptake into hepatocytes (Slc10a1 and Abcc4) and synthesis of neutral and hydrophobic bile acid (Cyp8b1 and Cyp7a1) were measured. Data represent levels of transcripts in each genotype liver relative to those in control liver and are expressed as means; error bars denote SEM. *p < 0.05; **p < 0.01 (n = 4 each genotype). (DOCX) [file pgen.1005401.s004.docx]

**S4 Fig. Aberrant morphology of hepatocytes and injury in DKO liver.**

(A and B) H&E stained section (A) and immunohistochemical analysis (B) on representative liver paraffin sections from 2-week-old mice. Scale bars, 50 μm. (C) H&E stained sections of liver from 7-week-old control and DKO. Scale bars, 200 μm. (D) Serum level analysis of asparate aminotransferase (AST), alanine aminotransferase (ALT), alkaline phosphatase (ALP), γ-glutamyltranspeptidase (GTP), total cholesterol, total bilirubin and total bile acid in 3­–6-week-old mice. Results are shown as mean±SEM. *p < 0.05; **p < 0.01 (n = 4 each genotype). (E) Real-time RT-PCR was performed to measure the expression of transcripts encoding bile acid synthesis and transport pathways in the livers of 3­–6-week-old control and DKO mice. Genes involved in bile acid efflux and uptake into hepatocytes (*Slc10a1* and *Abcc4*) and synthesis of neutral and hydrophobic bile acid (*Cyp8b1* and *Cyp7a1*) were measured. Data represent levels of transcripts in each genotype liver relative to those in control liver and are expressed as means; error bars denote SEM. *p < 0.05; **p < 0.01 (n = 4 each genotype).
