# Supplementary material for: Redundant Roles of Rpn10 and Rpn13 in Recognition of Ubiquitinated Proteins and Cellular Homeostasis
Source: PLoS Genet. 2015 Jul 29;11(7):e1005401. doi: 10.1371/journal.pgen.1005401 (PMC4519129; doi:10.1371/journal.pgen.1005401)
Supplement: S5 Fig — Immunoblot analysis of whole-cell extracts of livers from 2–4-week-old control and DKO with antibodies against indicated proteins. (DOCX) [file pgen.1005401.s005.docx]

**S5 Fig. Synthetic effect between Rpn10-UIM and Rpn13 on ubiquitin mediated protein degradation.**

Immunoblot analysis of whole-cell extracts of livers from 2-4-week-old control and DKO with antibodies against indicated proteins.
