# Supplementary material for: Redundant Roles of Rpn10 and Rpn13 in Recognition of Ubiquitinated Proteins and Cellular Homeostasis
Source: PLoS Genet. 2015 Jul 29;11(7):e1005401. doi: 10.1371/journal.pgen.1005401 (PMC4519129; doi:10.1371/journal.pgen.1005401)
Supplement: S1 Table — Primers a, b, and c were used for Rpn13 flox mice genotyping. Primers a and d were used for Rpn13 knockout mice genotyping. (DOCX) [file pgen.1005401.s006.docx]

**S1 Table. PCR primers for genotyping.**

Primers a, b, and c were used for Rpn13 flox mice genotyping. Primers a and d were used for Rpn13 knockout mice genotyping.

| Primer name | Sequence (5'-3') |
| --- | --- |
| a | GTTCTGGTCACCTGAGCACAAGGATC |
| b | TCCTGCCAGAGAGCATATGC |
| c | GAGAACCTGCGTGCAATCCATCTTG |
| d | TAGCCACAAGAGGCTGTAGTGACCAC |
|  |  |
| Primer a, b, and c were used for Rpn13 flox mice genotyping | |
| Primer a and d were used for Rpn13 knockout mice genotyping | |
