# Supplementary material for: Redundant Roles of Rpn10 and Rpn13 in Recognition of Ubiquitinated Proteins and Cellular Homeostasis
Source: PLoS Genet. 2015 Jul 29;11(7):e1005401. doi: 10.1371/journal.pgen.1005401 (PMC4519129; doi:10.1371/journal.pgen.1005401)
Supplement: S2 Table — (DOCX) [file pgen.1005401.s007.docx]

**S2 Table. PCR primers and universal probes for real time PCR.**

| Gene | Probe number | Forward primer (5'-3') | Reverse primer (5'-3') |
| --- | --- | --- | --- |
| *Gusb* | 42 | CTCTGGTGGCCTTACCTGAT | CAGTTGTTGTCACCTTCACCTC |
| *Slc10a1* | 78 | TGAAGGGGGACATGAACCT | GTAGATGTATAAGAGGAGAGGCATCA |
| *Abcc4* | 60 | CCACATGATTTACCGGAAGG | AGGTTAACTATCTGGCCTGTGG |
| *Cyp8b1* | 60 | CAGGAAGTTCCGTCGATTTG | GGCCCCAGTAGGGAGTAGAC |
| *Cyp7a1* | 92 | CACCATTCCTGCAACCTTCT | TTGGCCAGCACTCTGTAATG |
| *Noxa (Pmaip1)* | 15 | CAGATGCCTGGGAAGTCG | TGAGCACACTCGTCCTTCAA |
| *Puma (Bbc3)* | 79 | TTCTCCGGAGTGTTCATGC | TACAGCGGAGGGCATCAG |
| *Gpc3* | 17 | CGGTGGTTAGCCAGATCATT | CTTGGGCACAGACATGGTT |
| *Afp* | 63 | CATGCTGCAAAGCTGACAA | CTTTGCAATGGATGCTCTCTT |
| *Col1a1* | 18 | AATGGCGATCGTGGTGAG | GCCTGTCTCACCCTTGTCA |
| *Epcam* | 53 | GGTTAGCGCTTCCGAGGTA | TGTTGGATAGTCAAGGCCAGT |
| *α6 (Psma1)* | 73 | GACCACAGAGAAAAGCACAGC | ATTGGTTCATCGGCTTTTTC |
| *Rpn6 (Psmd11)* | 5 | CGGTCTCTTCTTGATCTGTTTCTA | GATGCACTCTAAACATAGCTCGAC |
| *Rpt4 (Psmc6)* | 72 | CGTTGTAGGTTGTCGTCGTC | TGTGGTCATATCCAAAGCAACT |
| *Nrf1 (Nfe2l1)* | 82 | GCAGCTTGGTCTCATCCAG | CAGGTCTTTGTCTTGGGTCAG |
| *p62 (Sqstm1)* | 63 | TTGGAGTCGGTGGGACAG | TGAAGACAAATGTGTCCAGTCAT |
| *Ho-1 (Hmox1)* | 17 | AGGCTAAGACCGCCTTCCT | TGTGTTCCTCTGTCAGCATCA |
| *Bip (Hspa5)* | 105 | CTGAGGCGTATTTGGGAAAG | TCATGACATTCAGTCCAGCAA |
| *Chop (Ddit3)* | 73 | AAGCATGTGACCCTGCACT | TCCTGGTCTACCCTCAGTCC |
| *β-catenin (Ctnnb1)* | 93 | TTCCTATGGGAACAGTCGAAG | TTGTATTGTTACTCCTCGACCAAA |
